# Virulence-Dependent Alterations in the Kinetics of Immune Cells during Pulmonary Infection by *Mycobacterium tuberculosis*

**DOI:** 10.1371/journal.pone.0145234

**Published:** 2015-12-16

**Authors:** Woo Sik Kim, Jong-Seok Kim, Seung Bin Cha, Seung Jung Han, HongMin Kim, Kee Woong Kwon, So Jeong Kim, Seok-Yong Eum, Sang-Nae Cho, Sung Jae Shin

**Affiliations:** 1 Department of Microbiology, Institute for Immunology and Immunological Diseases, Yonsei University College of Medicine, Seoul, South Korea; 2 Brain Korea 21 PLUS Project for Medical Science, Yonsei University College of Medicine, Seoul, South Korea; 3 Division of Immunopathology and Cellular Immunology, International Tuberculosis Research Center, Changwon, South Korea; Public Health Research Institute at RBHS, UNITED STATES

## Abstract

A better understanding of the kinetics of accumulated immune cells that are involved in pathophysiology during *Mycobacterium tuberculosis* (Mtb) infection may help to facilitate the development of vaccines and immunological interventions. However, the kinetics of innate and adaptive cells that are associated with pathogenesis during Mtb infection and their relationship to Mtb virulence are not clearly understood. In this study, we used a mouse model to compare the bacterial burden, inflammation and kinetics of immune cells during aerogenic infection in the lung between laboratory-adapted strains (Mtb H37Rv and H37Ra) and Mtb K strain, a hyper-virulent W-Beijing lineage strain. The Mtb K strain multiplied more than 10- and 3.54-fold more rapidly than H37Ra and H37Rv, respectively, during the early stage of infection (at 28 days post-infection) and resulted in exacerbated lung pathology at 56 to 112 days post-infection. Similar numbers of innate immune cells had infiltrated, regardless of the strain, by 14 days post-infection. High, time-dependent frequencies of F4/80^-^CD11c^+^CD11b^-^Siglec-H^+^PDCA-1^+^ plasmacytoid DCs and CD11c^-^CD11b^+^Gr-1^int^ cells were observed in the lungs of mice that were infected with the Mtb K strain. Regarding adaptive immunity, Th1 and Th17 T cells that express T-bet and RORγt, respectively, significantly increased in the lungs that were infected with the laboratory-adapted strains, and the population of CD4^+^CD25^+^Foxp3^+^ regulatory T cells was remarkably increased at 112 days post-infection in the lungs of mice that were infected with the K strain. Collectively, our findings indicate that the highly virulent Mtb K strain may trigger the accumulation of pDCs and Gr1^int^CD11b^+^ cells with the concomitant down-regulation of the Th1 response and the maintenance of an up-regulated Th2 response without inducing a Th17 response during chronic infection. These results will help to determine which immune system components must be considered for the development of tuberculosis (TB) vaccines and immunological interventions.

## Introduction

*Mycobacterium tuberculosis* (Mtb) causes tuberculosis (TB) and leads to the most infectious bacteria-related mortalities in the world [1]. In 2014, there were 8.6 million new cases of TB and 1.3 million deaths from TB that were reported by the World Health Organization, indicating that improved treatment and prevention strategies are urgently needed [1]. Because only approximately 5 to 10% of immunocompetent individuals develop active TB during their lifetimes, host immune status is considered to be a major factor driving TB infection [2]. However, current TB pathogenesis paradigms are changing with respect to pathogen diversity because more virulence has been identified in Mtb clinical isolates than was previously anticipated [3]. Recently, the paradigm has shifted to focus on understanding the immunology of and granuloma formation in primary and post-primary TB [4]. For example, virulent Mtb releases large amounts of trehalose-6,6’-dimycolate (TDM; also known as cord factor) during growth [4]. Cord factor could influence granuloma development after the adaptive transfer of CD4^+^ T cells from TDM-immunized mice, which could provide a better understanding of TB pathogenesis in terms of cellular immunity [4, 5].

To date, most virulence studies of different Mtb strains have focused on the laboratory-adapted reference strains Mtb H37Rv and H37Ra, which are virulent and attenuated strains, respectively [6, 7]. In addition, many studies have used a specific genetic knockout mouse to investigate TB pathogenesis [8–10]. However, there have not been studies to investigate the effect of switching the immune cell population and to examine the resulting virulence of Mtb strains, including clinical isolates in immunocompetent conditions. Most of the previous studies investigated immune-related factors at specific time point post-infection [11–13]. It is important to directly study the events occurring *in vivo* from very early time points to late time-points. It is also important to investigate the increases or decreases of specific cell populations during lung infections in a time- and virulence-dependent manner, including those of innate cells and T cells.

Mtb strains in different populations or geographical locations can exhibit different levels of virulence during the human-adaptation process, with consequent varying epidemiological dominance (e.g., Beijing and Euro-American Haarlem) [14, 15]. Importantly, clinical and epidemiological studies have shown that the emergence of the Beijing strains may be associated with multi-drug resistance and a high level of virulence, resulting in increased transmissibility and rapid progression from infection to active disease [16, 17]. In addition, Mtb belonging to the Beijing genotypes induces more disease progression and relapse from the latent state [16, 17]. For example, HN878 belongs to the Beijing lineage, and it has been the causative agent of major TB outbreaks. It causes rapid disease progression to death in comparison to other clinical isolates (CDC1551) and to the reference virulent strain H37Rv [18, 19]. In addition, a low-dose infection of C57BL/6 mice with Mtb strain 1471 of the Beijing genotype family caused the rapid development of extensive granulomatous inflammation with necrotic areas and intense bacillus dissemination, rapidly leading to animal death [20]. However, the exact pathogenesis of Beijing isolates is inconclusive, and the isolates demonstrate a wide range of inflammatory and virulence phenotypes, as determined in animal models and in *in vitro* models of macrophage infection [20]. Factors that contribute to the pathogenesis of the Beijing genotype with respect to the host immune response remain largely unknown.

The Mtb K strain phylogenetically belongs to the Beijing genotype; it was most widely isolated in an outbreak of pulmonary TB in senior high schools in South Korea [21]. Importantly, our previous study showed that Mtb K replicated rapidly during the early stages of infection in a murine model of TB with a more severe pathology [22]. During the study, intriguingly, we observed that there was only a small colony forming unit (CFU) difference at later time points (> 12 weeks post-infection) between the K strain and H37Rv, but the K strain led to death earlier than H37Rv because of an excessive host immune response [22]. Thus, the immunopathology that was induced by hypervirulent Mtb or by the host immune responses during lung infection led us to investigate the kinetics of the host immune cell response in Mtb pathogenesis. In particular, early alterations in innate immune cells have attracted less attention than adaptive immune responses, such as those involving T cells. Thus, a better understanding of immune cell kinetics and of the immune response during lung infection in an Mtb virulence-dependent manner may facilitate the rational design of more effective therapeutic interventions, including immunotherapeutic vaccines.

To gain insight into the host response that culminates in the progression of infection to severe TB disease, we investigated immune cell kinetics from early (2 days) to late (112 days) time points post-infection with the Mtb H37Ra, H37Rv and K strains in the lungs of aerogenically infected mice. In addition, alterations to the immune cells and the consequent changes in pathological development in response to Mtb virulence were investigated during lung infection.

## Materials and Methods

### Ethics Statement

All animal experiments were performed in accordance with the Korean Food and Drug Administration (KFDA) guidelines. The experimental protocols used in this study were reviewed and approved by the Ethics Committee and Institutional Animal Care and Use Committee (Permit Number: 2014-0197-1) of the Laboratory Animal Research Center at Yonsei University College of Medicine (Seoul, Korea).

### *M*. *tuberculosis* strains and culture conditions

Mtb H37Rv (ATCC 27294) and H37Ra (ATCC 25177) were purchased from American Type Culture Collection (ATCC, Manassas, VA), and the Mtb K strain was obtained from the strain collections at the Korean Institute of Tuberculosis (Korean National Tuberculosis Association, Seoul, Republic of Korea). All strains were cultured in Middlebrook 7H9 broth (Difco Laboratories, Detroit, MI) supplemented with 0.02% glycerol and 10% (vol/vol) oleic acid-albumin-dextrose-catalase (OADC, Becton Dickinson, Sparks, MD) for 28 days at 37°C. Single cell suspensions of each strain were prepared as previously described with slight modifications. Briefly, mycobacterial cells grown in OADC-supplemented 7H9 broth were harvested by centrifugation at 10,000 × *g* for 20 min and washed three times in phosphate-buffered saline (PBS) (pH 7.2). The pellets were homogenized using an overhead stirrer (Wheaton Instrument, Millville, NJ) for 1 min on ice to minimize bacterial clumping. The homogenized mycobacterial cells were passed through an 8 μm filter (Millipore Corp., Bedford, MA). The predominant presence of single cells in the final preparation was confirmed by acid-fast staining. The seed lots of each strain were stored in small aliquots at -80°C until use. The CFUs per 1 ml of the seed lots were measured by a viable counting assay on 7H10 agar plates.

### Animals, aerosol infection, and bacterial load determination

Specific pathogen-free 5- to 6-week-old female C57BL/6 mice as well as OT-I and OT-II T-cell receptor (TCR) transgenic mice (C57BL/6 background) were purchased from the Jackson Laboratory (Bar Harbor, ME, USA). Mice were maintained under barrier conditions in a BL-3 biohazard animal facility at the Yonsei University Medical Research Center with constant temperature (24 ± 1°C) and humidity (50 ± 5%). Animals were fed a sterile commercial mouse diet with ad libitum access to water under standardized light-controlled conditions (12 h light and 12 h dark periods). Mice were monitored daily, and none of the mice exhibited any clinical symptoms or illnesses during this experiment.

For animal infection studies, mice were aerogenically infected with Mtb strains (H37Rv and H37Ra and Beijing-K) as previously described [22]. Non-infected naïve mice were used as a control for histopathology and flow cytometry analysis. Briefly, 70 mice per group were exposed to a predetermined dose of a Mtb strain for 60 min in the inhalation chamber of an airborne infection apparatus (Glas-Col, Terre Haute, IN) to achieve initial infectious doses of 450–500 CFU of H37Ra or 150–200 CFU of H37Rv or K per mouse lung (*n* = 4). Nine mice per group (4 mice for immune cell kinetics analysis and 5 mice for CFU and histopathology analyses) were euthanized at days 2, 5, 7, and 14 post-infection (early infection period) and days 28, 56, and 112 post-infection (late infection period). All animal experiments were conducted according to the ethical and experiment regulations of the Yonsei University Health System Institutional Animal Care and Use Committee.

At the indicated time points, the mice were humanely euthanized via CO_2_ inhalation, and the lungs were harvested for CFU enumeration, histology, and flow cytometry analysis. The numbers of viable bacteria in the lungs were determined by plating 5-fold serial dilutions of whole organ homogenates onto Middlebrook 7H10 agar (Becton Dickinson, Franklin Lakes, NJ, USA). Bacterial colonies were counted 8 weeks after incubation at 37°C. The data are reported as the median log_10_ CFU ± interquartile range (IQR).

### Histopathology and assessment of lung inflammation

Tissue samples were collected for histopathology, preserved in 10% neutral-buffered formalin, embedded in paraffin, cut into 5 to 6 μm sections, and stained with hematoxylin and eosin (H&E) or acid-fast stain as previously described [23]. The severity of the inflammation in the lungs of the mice was evaluated as previously described [23]. In brief, H&E-stained lung sections were photographed at × 40 magnification using a microscope (Olympus, Tokyo, Japan BX51), and granulomas were analyzed relative to the lung area with image analysis software (ImageJ, National Institutes of Health, USA) to obtain the affected percentage of the lung area. Ten lesions from each group were randomly selected and analyzed.

### Lung cell preparation

The lung cells were prepared under sterile conditions as previously described. Briefly, the lungs were cut into 0.5 cm pieces and agitated in 5 ml of cellular dissociation buffer (Roswell Park Memorial Institute (RPMI) medium (Biowest, Nuaillé, France) containing 0.1% collagenase type IV (Worthington Biochemical Corporation, NJ, USA), 1 mM CaCl_2_, and 1 mM MgCl_2_) for 15 min at 37°C. Then, the lung cells and aggregates were filtered through a 40 μm cell strainer (BD Biosciences) in Dulbecco’s PBS using a sterile 1 ml syringe. The erythrocytes were lysed using red blood cell lysis buffer (90 ml of 0.16 M NH_4_ and 10 ml of 0.17 M Tris, pH 7.65) for 2 min at room temperature, and the lung cells were washed twice with RPMI 1640 medium supplemented with 2% fetal bovine serum (Biowest, Nuaillé, France).

### Antibodies

Single-cell suspensions of lungs from naïve- and Mtb-infected mice were stained with primary fluorochrome-conjugated antibodies. Negative samples were stained with isotype control antibodies that were conjugated to the same fluorochrome. The following reagents were obtained from eBioscience (San Diego, CA): anti-CD11c (N418, PE-Cy7), anti-CD11b (M1/70, FITC), anti-F4/80 (BM8, APC), anti-Gr-1 (RB6-8C5, PE), anti-Siglec-H (eBio440c, e450), anti-PDCA-1 (eBio927, PerCp-Cy5.5), anti-CD3e (145-2C11, eFluor 450), anti-T-bet (4B10, PE-Cy7), anti-RORγt (B2D, PE), anti-GATA-3 (L50-823, Alexa 647), anti-Foxp3 (NRRF-30, PE), anti-Rat IgG1 isotype control (eBRG1, PE), anti-Rat IgG2a isotype control (eBR2a, APC) and anti-Mouse IgG1 isotype control (M1-14D12, PE-Cy7). The following reagents were obtained from BD Bioscience (San Diego, CA): anti-CD16/32 (FcR III/II receptor, 2.4G2), anti-CD4 (RM4-5, PerCp-Cy5.5), anti-CD8 (53–6.7, APC-Cy7) and anti-CD25 (PC61, APC).

### Analysis of lung cell populations by flow cytometry

The phenotypes of pulmonary subpopulations were analyzed in single-cell suspensions. Cells (2 × 10^6^/ml) were first blocked with anti-CD16/32 for 20 min at 4°C, and then the cell surface was stained with fluorochrome-conjugated mAbs (lung infiltrating cell analysis: CD11c, CD11b, F4/80, Gr-1, Siglec-H, and PDCA-1; T cell analysis: CD3, CD4, CD8 and CD25) for 30 min at 4°C. Transcription factor-analyzed cells were either fixed or permeabilized with the transcription factor staining kit (eBioscience). They were then incubated for 30 min at 4°C and intracellularly stained with T cell-specific fluorochrome-conjugated mAbs against T-bet, RORγt, GATA-3 and Foxp3 for 30 min at 4°C. Rat IgG1, Rat IgG2a and Mouse IgG1 were used as the isotype controls. We obtained the absolute number of each cell type by multiplying the percentage of each cell type by the total number of cells in the single-cell suspension from the lungs of Mtb-infected mice. The cells were analyzed using a FACSVerse flow cytometer (BD bioscience) and FlowJo software (BD Bioscience).

### Cytokine measurements in *ex vivo* lung cells culture after PPD stimulation

Single-cell suspensions from the lungs of Mtb-infected mice (at days 2, 5, 7, 14, 28, 56 and 112 post-infection) were stimulated with purified protein derivative (PPD; 10 μg/ml), which was kindly provided by Dr. Brennan at Aeras (Rockville, MD), for 24 h at 37°C. IL-5, IL-17A, IL-10 (eBioscience), IFN-γ and IFN-α (PBL Assay Science, Piscataway, NJ, USA) cytokine levels were analyzed in the culture supernatant by ELISA according to the manufacturer’s protocol.

### Generation and culture of bone marrow-derived DCs

Bone marrow-derived DCs (BMDCs) were prepared and cultured as described recently [24]. On day 8, over 80% of the nonadherent cells expressed CD11c. To obtain highly purified populations for subsequent analyses, the DCs were labeled with a bead-conjugated anti-CD11c mAb (Miltenyi Biotec, San Diego, CA), followed by positive selection on paramagnetic columns (LS columns; Miltenyi Biotec) according to the manufacturer’s instructions. The purity of the selected cell fraction was > 90%.

### Isolation of innate cells from Mtb-infected lungs

Single-cell suspensions of lung cells were enriched for plasmacytoid DCs (pDCs) using the Plasmacytoid DC Isolation Kit II according to the manufacturer’s instructions with slight modifications. Briefly, lung cells were blocked with anti-CD16/32 at 4°C for 20 min, labeled with bead-conjugated anti-CD11b and CD3 mAbs, and separated by sequential passage through LS MACS columns. The negative fractions were stained with a pDC Biotin Cocktail, incubated with anti-Biotin MicroBeads, and separated by sequential passage through LS MACS columns. To isolate CD11c^-^/CD11b^+^/Gr-1^int^ cells, single-cell suspensions of lung cells were stained with a mixture of bead-conjugated anti-CD4, CD8, Gr-1, CD11c, and B220 mAbs and separated by sequential passaging through LS MACS columns. The negative fractions were stained with CD11b MicroBeads and separated by sequential passaging through LS MACS columns. To isolate CD11b^high^ DCs, single-cell suspensions were stained with bead-conjugated anti-CD11b mAb and separated by sequential passages through LS MACS columns. Next, the CD11b^+^ fraction was labeled with a bead-conjugated anti-CD11c mAb and separated by sequential passaging through LS MACS columns. The purity of the selected pDC, Gr-1^int^ cell and CD11b^high^ DC fractions was >70%. Columns and antibodies for isolation of innate cells purchased from Miltenyi Biotec (San Diego, CA).

### Functional analysis of isolated innate cells with T cells

To analyze the function of DCs sorted from Mtb-infected mice, isolated CD11b^high^ DCs were treated for 1 h with OVA peptides (CD4^+^ specific OVA_323-339_ or CD8^+^ specific OVA_257-264_) synthesized by AbFrontier (Seoul, Korea). CD4^+^ and CD8^+^ T cells were isolated from splenocytes prepared from OT-II and OT-I mice using CD4 and CD8 T cell isolation kits (Miltenyi Biotec), respectively. These T cells were stained with 1 μM CFSE (Invitrogen). Next, OVA-treated CD11b^high^ DCs (1 × 10^5^ cells per well) were co-cultured with CFSE-stained CD8^+^ and CD4^+^ T cells (1 × 10^6^) at an isolated cell:T cell ratio of 1:10. To analyze the function of pDCs and CD11c^-^/CD11b^+^/Gr-1^int^ cells sorted from Mtb-infected mice, isolated cells (pDCs and CD11c^-^/CD11b^+^/Gr-1^int^ cells) with or without BMDCs were co-cultured with CD4^+^ or CD8^+^ T cells from OT-I and OT-II mice. For the group with BMDCs, the BMDCs were treated with OVA peptides. For the group without BMDCs, the OVA peptides were directly administered to the isolated cells (pDCs and CD11c^-^/CD11b^+^/Gr-1^int^ cells). After 1 h incubation, the OVA-treated cells were washed with PBS and then co-cultured with CD4^+^ and CD8^+^ T cells from OT-I or OT-II mice. The ratio of T cells to isolated cells was 10:1, and for the group with BMDCs, the ratio between T cells, isolated cells, and BMDCs was 1:1:10. After 3 days of co-culture, the T cells were stained with anti-CD4 and anti-CD8 antibodies and analyzed using a FACSVerse flow cytometer and FlowJo software. Supernatants were harvested, and cytokine production was analyzed (IL-17A, IL-5, IL-2 and IFN-γ; eBioscience) by ELISA according to the manufacturer’s protocol.

### Statistical analysis

The data are reported as the median ± interquartile range (IQR) or the mean ± standard deviation (SD). The levels of significance for comparisons between samples were determined by the Kruskal-Wallis test followed by Dunn’s test using statistical software (GraphPad Prism Software V5.0; GraphPad Software, San Diego, CA, USA). Values of *p < 0.05, **p < 0.01 and ***p < 0.001 were considered significant.

## Results

### Course of infection in the lungs of mice infected with different virulent strains of *M*. *tuberculosis*

**Fig 1:**
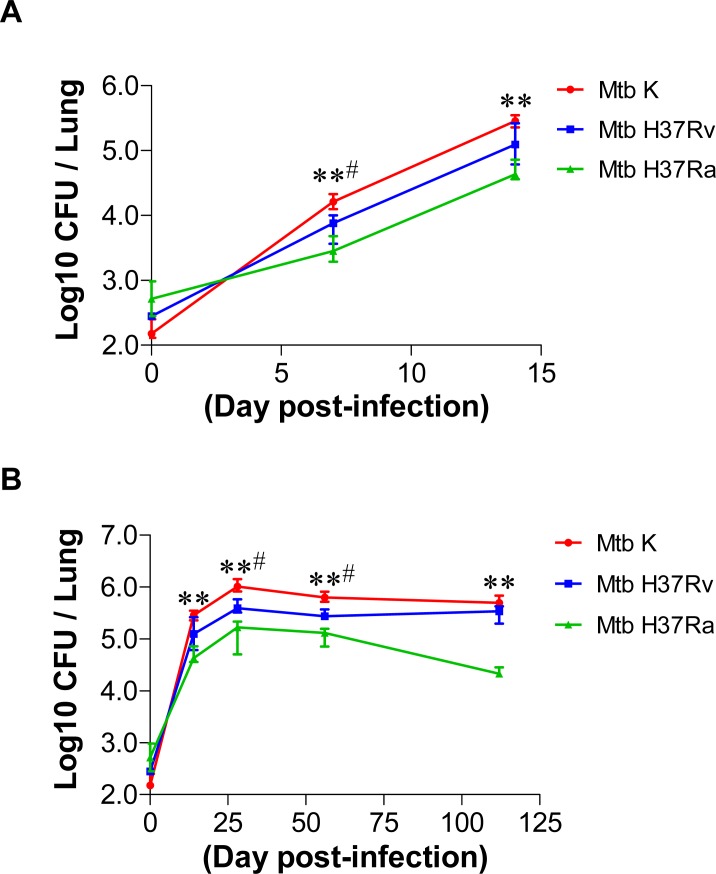
Comparative growth profiles of tested Mtb strains in the lungs during a 112 day-infection. (A) Early change in Mtb growth during the first 14 days of infection in C57BL/6 mice. (B) The overall growth pattern of tested Mtb strains in C57BL/6 mice. Mice (*n* = 5 per group at each designated time point) were aerosol challenged with approximately 450 CFU of Mtb H37Ra or with approximately 200 CFU of the H37Rv or the Beijing-K strain. The bacterial burden of their lungs was determined at various time points post-infection. Data are presented as the median log_10_ CFU of five mice (± IQR) for each time point. A *p*-value ≤ 0.05 was considered significant: **p < 0.01 (Mtb K *vs*. Mtb H37Ra) and ^#^ *p* < 0.05 (Mtb K *vs*. Mtb H37Rv).

In this study, we examined the bacterial burdens in the lung during early (by 14 days post-infection) and late infection (14–112 days post-infection) according to Mtb virulence (Fig 1). After the aerosol challenge, the bacterial CFU of Mtb K increased approximately 2.5- and 5.5-fold faster at 7 days post-infection than the CFU of Mtb H37Rv (*p* < 0.05) or H37Ra (*p* < 0.01), respectively. At 14 days post-infection, Mtb K grew approximately 2.3- and 5.9-fold faster than Mtb H37Rv (*p* < 0.05) and H37Ra (*p* < 0.01), respectively (Fig 1A). At 28 and 56 days post-infection, a significantly higher Mtb K burden was present (*p* < 0.05 *vs*. H37Rv and *p* < 0.01 *vs*. H37Ra). The bacterial CFU of each strain in the lungs peaked at 28 days post-infection, and the peaked CFU was maintained through 112 days post-infection. The CFU values of Mtb K and H37Rv approached similar levels at 112 days post-infection (Fig 1B). Interestingly, the bacterial CFU level in the lungs of mice infected with Mtb H37Ra started to decline after 28 days, and lower bacteria CFU was observed at 112 days post-infection than at 14 days post-infection.

### Lung pathology after aerosol infection with different virulent *M*. *tuberculosis* strains

**Fig 2:**
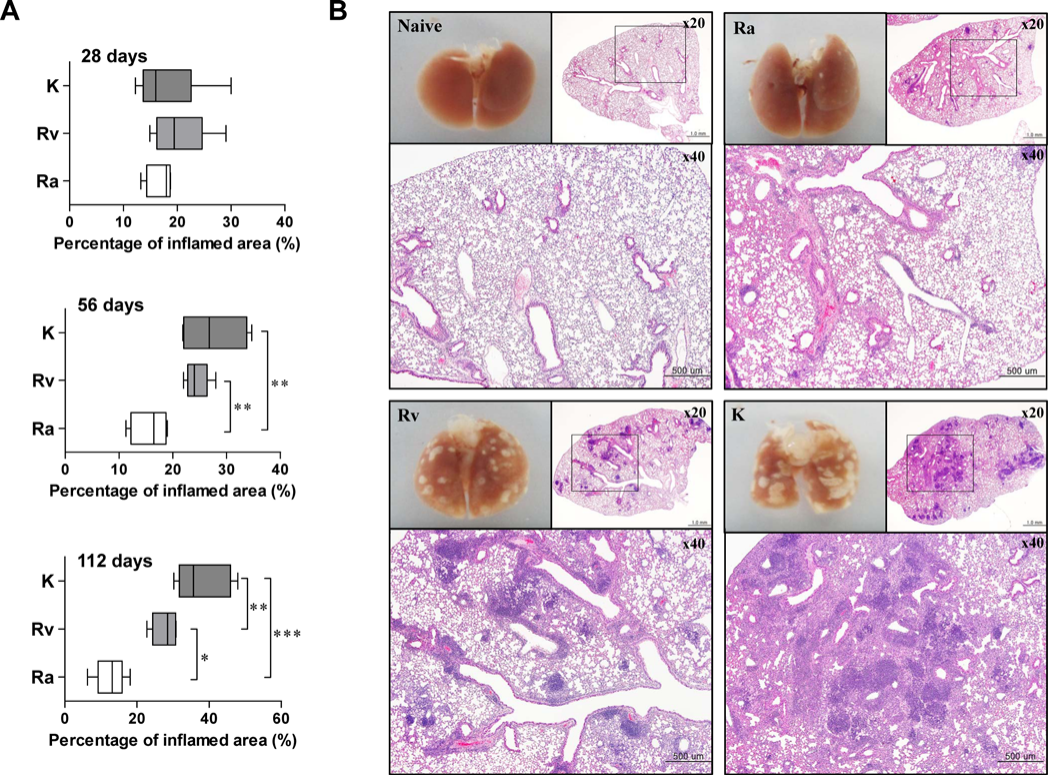
Histopathological analysis of lungs infected with different strains of virulent Mtb. (A) Inflammatory scores of H&E-stained sections of lungs during Mtb infection. At 28, 56, and 112 days post-infection, mice were sacrificed, and lung sections were stained with H&E (*n* = 5 per group per designated time point). Ten pictures from each group were randomly selected and analyzed (2 pictures per mouse × 5 mice in each group). Lung inflammation scores are presented as the median percentage (± IQR) of inflammation for each mouse. Lung sections were stained with H&E (bar, 500 μm). A *p*-value ≤ 0.05 was considered significant: *p < 0.05, **p < 0.01 and ***p < 0.001. (B) Representative histopathology and gross pathology of mouse lungs infected with Mtb strains with different virulence levels at 112 days post-infection.

The gross pathology and histopathology of lungs were compared after aerosol infection with different virulent Mtb strains (Fig 2, S1 Fig and S2 Fig), and representative lung section histopathology shows the median percentage of inflammation at 112 days post-infection (Fig 2B). Granulomatous inflammations in the lungs began to appear at 28 days post-infection in all of the tested strains, but there were no significant differences in the level of lung inflammation among the 3 groups at 28 days post-infection (Fig 2A, upper panel, and S1 Fig). However, the levels of granulomatous inflammation in Mtb K- and H37Rv-infected mice were significantly higher than those in Mtb H37Ra-infected mice at 56 days post-infection (*p* < 0.01; Fig 2A, middle panel, and S1 Fig). Lung inflammation was significantly greater in Mtb K-infected mice than in either Mtb H37Ra- (*p* < 0.001) or Mtb H37Rv-infected mice *(p* < 0.01) at 112 days post-infection (Fig 2A, lower panel). Interestingly, severe immunopathology and extensive granulomatous inflammation were only clearly observed in the lungs of mice that were infected with Mtb K at 112 days post-infection (Fig 2B and S2 Fig).

### Comparative analysis of the kinetics of innate immune cell populations during lung infection with *M*. *tuberculosis*

**Fig 3:**
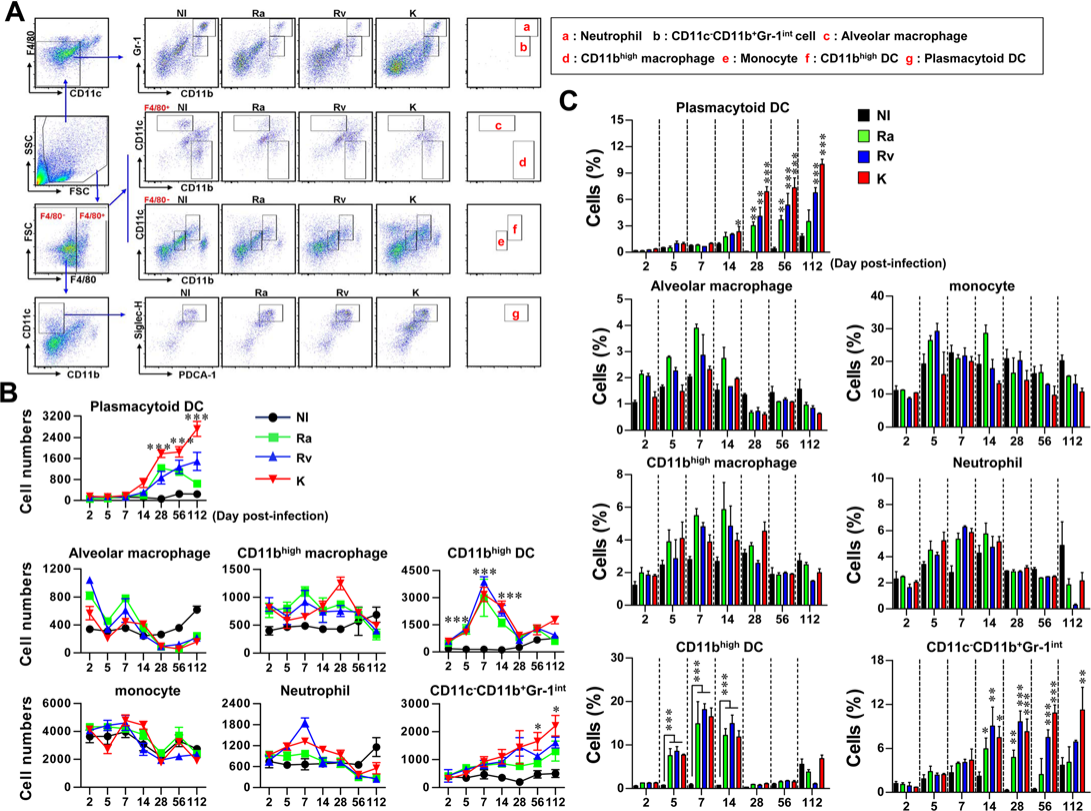
Analysis of innate immune cell kinetics of mice infected with Mtb with different virulence levels. Single-cell suspensions prepared from lung tissues of mice infected with Mtb strains at days 2, 5, 7, 14, 56 and 112 days post-infection were stained with the indicated antibodies and analyzed by flow cytometry (*n* = 4 per group per designated time point). (A) Gating strategy for the analysis of innate immune cells present in the in lungs. All surface-stained samples were primarily gated on forward scatter (FSC)^mid^/^high^ and side scatter (SSC)^mid^/^high^ and secondarily gated on F4/80^-^/^+^ and CD11c^low^. The cells were analyzed according to the expression of Gr-1 versus CD11b. F4/80^-^/CD11c^-^/CD11b^high^/Gr-1^high^ cells were designated as neutrophils (a). The lower population was designated as CD11c^-^/CD11b^+^/Gr-1^int^ cells (b). Next, dot-blots of lung cells were primarily gated on FSC^mid^/^high^ and SSC^mid^/^high^ and secondarily gated on F4/80^-^ and F4/80^+^. The cells were analyzed according to the expression of CD11b versus CD11c. F4/80^+^/CD11c^+^/CD11b^-^ cells (c), F4/80^+^/CD11c^-^/CD11b^+^ cells (d), F4/80^-^/CD11c^int^/CD11b^int^ cells (e), F4/80^-^/CD11c^+^/CD11b^+^ cells (f) and F4/80^-^/CD11c^+^/CD11b^-^/Siglec-H^+^/PDCA-1^+^ cells (g) were designated as alveolar macrophages, CD11b^+^ macrophages, monocytes, CD11b^high^ DCs and pDCs, respectively. (B) The line graphs display the absolute numbers of cells in (A) at various time points during Mtb lung infection. *p < 0.05, ***p < 0.001, Mtb K-infected *vs*. Mtb H37Rv-infected groups. (C) Bar graphs shows the percentage of infiltrated cells in the lungs. *p < 0.05, **p < 0.01, ***p < 0.001, non-infected *vs*. Mtb-infected groups. The data are presented as the mean (± SD) or four mice per group at each time point from one representative experiment out of two independent experiments. NI: Non-infected; Ra: H37Ra-infected lung; Rv: H37Rv-infected lung; K: Beijing-K-infected lung.

During Mtb infection, it is important to understand the immune cell populations that are involved in the pathogenesis and regulation of the pathological immune response. However, there have been fewer studies on innate immune cells than on T cells. Thus, we investigated the influx of innate immune cells into the lung following infection with different strains of virulent Mtb. The kinetics of the infiltrating innate immune cells (neutrophils, monocytes, macrophages, CD11c^-^CD11b^+^Gr-1^int^ cells, CD11b^high^ DCs and pDCs) were investigated upon infection with Mtb (Fig 3A). F4/80^-^CD11b^high^CD11c^+^ DCs (CD11b^high^ DCs) were observed during the early infection period (before 14 days post-infection) of Mtb strains (*p* < 0.001 at 5, 7 and 14 days post-infection) (Fig 3B and 3C). In addition, higher numbers of F4/80^-^CD11b^-^CD11c^+^Siglec-H^+^PDCA-1^+^ DCs (pDCs) (*p* < 0.001 at 28, 56 and 112 days post-infection) and CD11c^-^CD11b^+^Gr-1^int^ cells (*p* < 0.05 at 56 and 112 days post-infection) were observed in a time-dependent manner only in the lungs of mice that were infected with Mtb K (Fig 3B). Importantly, higher frequencies of pDCs and CD11c^-^CD11b^+^Gr-1^int^ cells were observed in all Mtb strain-infected mice after 28 days post-infection (Fig 3C).

### Alteration of T cell subtypes and polarization in the lung during *M*. *tuberculosis* infection

**Fig 4:**
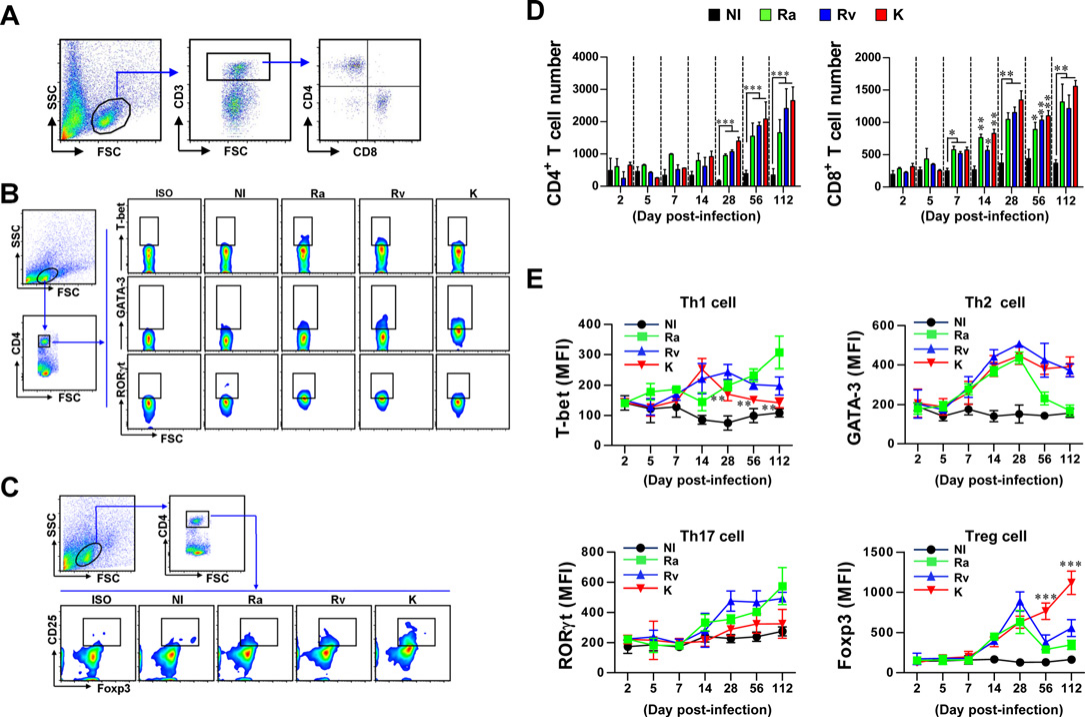
Analysis of T cell kinetics, subtypes, and polarization in Mtb-infected mice. Single-cell suspensions prepared from lung tissues of mice infected with Mtb strains at 2, 5, 7, 14, 28, 56 and 112 days post-infection. A representative gating strategy (at 112 days post-infection) for the assessment of CD4 T cells, CD8 T cells, Th1, Th2, Th17, and Treg cells is shown in the left panel of (A), (B), and (C). CD4^+^ and CD8^+^ T cells were stained with the indicated surface and transcription factor antibodies and analyzed by flow cytometry. (D) Bar graphs display the absolute numbers of CD3^+^/CD4^+^ and CD3^+^/CD8^+^ T cells in the lungs at 2, 5, 7, 14, 28, 56 and 112 days post-infection. (E) Using CD3^+^CD4^+^ T cells as the parent gate, specific staining for the transcription factors T-bet, GATA-3, RORγt and Foxp3 in lung cells from Mtb-infected mice is shown at various time points. Line graphs show the expression of T-bet (Th1 cells), GATA-3 (Th2 cells), RORγt (Th17 cells) and Foxp3 (Tregs) in CD3^+^/CD4^+^ T cell populations at the indicated time points. *p < 0.05, **p < 0.01, and ***p < 0.001, Mtb K-infected group *vs*. Mtb H37Rv-infected group. One representative plot out of two independent experiments is shown.

**Fig 5:**
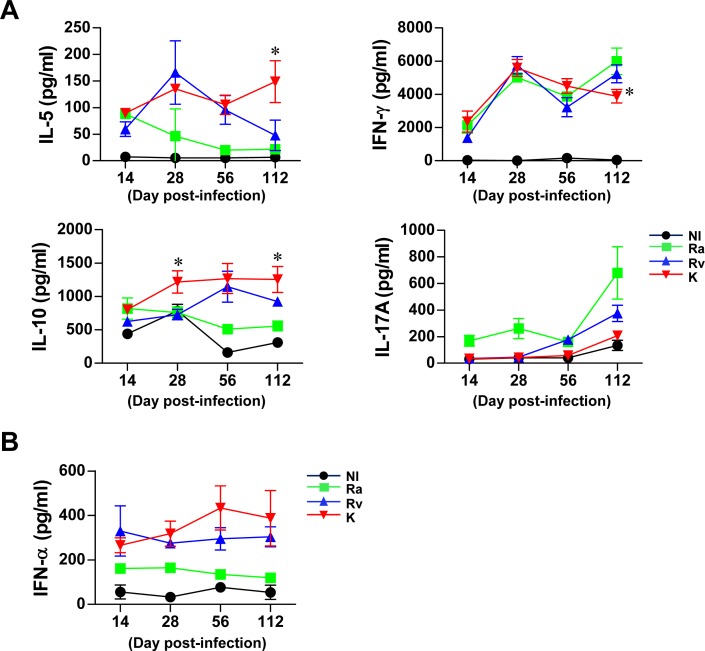
PPD-specific cytokine response in lung cells from Mtb-infected mice. The amount of IFN-γ, IL-5, IL-10, IL-17A (A) and IFN-α (B) produced by lung cells (14, 28, 56 and 112 days post-infection) in response to PPD (10 μg/ml) stimulation for 24 h was measured by ELISA. All data are expressed as the mean ± SD (*n* = 4 per group per designated time point) of one representative experiment out of two independent experiments.

Mtb-induced T cells play a key role in granuloma formation by regulating host-favored or pathogen-favored conditions. In addition, the initiation and maintenance of T cell subtypes will be crucial for future vaccine development. Thus, we analyzed the number, subtype and polarizing cytokines of T cells according to the virulence of the different Mtb strains (Fig 4 and Fig 5). Interestingly, CD4^+^ and CD8^+^ T cell numbers increased in a time-dependent manner for all of the Mtb strains. There were no significant differences between Mtb strains regarding CD4^+^ and CD8^+^ T cell numbers at any time point (Fig 4D). Next, we analyzed the time-dependent initiation, maintenance, and polarization of T cell subtypes according to the Mtb strain (Fig 4B, 4C and 4E). T-bet-expressing CD4^+^ T cells, representing Th1-type T cell immunity, increased in the lungs of mice that were infected with Mtb strains at 14 days post-infection in a time-dependent manner. However, this T cell subtype rapidly decreased in the lungs of mice infected with Mtb K after 14 days (*p* < 0.01 at 28, 56 and 112 days post-infection) (Fig 4E, upper-left panel). Interestingly, GATA-3-expressing CD4^+^ T cells, which are considered Th2-type T cells, accumulated more quickly and to higher levels than T-bet-expressing CD4^+^ T cells, regardless of the Mtb strain, and the levels peaked at 28 days post-infection. Furthermore, GATA-3-expressing CD4^+^ T cells started to decrease after 28 days post-infection, but their levels were maintained in the lungs of mice infected with both virulent Mtb K and H37Rv (Fig 4E, upper-right panel). RORγt-expressing CD4^+^ T cells, which are thought to be Th17-type T cells, were only present in the lungs of mice infected with the reference strains, Mtb H37Ra and H37Rv, at 28, 56 and 112 days post-infection; this type of T cell was not induced in mice infected with Mtb K (Fig 4E, lower-left panel). CD25^+^Foxp3^+^CD4^+^ Tregs increased from 7 days post-infection until 28 days post-infection in the lungs for all Mtb strains. Interestingly, Tregs gradually decreased in the lungs of mice infected with the reference strains but continually increased in a time-dependent manner in the lungs of mice infected with Mtb K (*p <* 0.001 at 56 and 112 days post-infection) (Fig 4E, lower-right panel).

### Cytokine production in *ex vivo* lung cell cultures after PPD stimulation

Along with alterations in innate cells and T cells, we also investigated cytokine production by lung cells after PPD stimulation (Fig 5). After 14 days post-infection, Th2 cytokines (IL-10 and IL-5) increased with PPD stimulation in lung cells from Mtb K-infected mice compared to those from Mtb H37Rv-infected mice, and Th1 cytokine production decreased at 112 days post-infection in Mtb K-infected mice compared to Mtb H37Rv-infected mice. In addition, IL-17 was not induced in Mtb K-infected mice compared to non-infected mice (Fig 5A). Before 14 days post-infection, there were no significant differences in these cytokines between mice infected with Mtb H37Ra and those infected with the two virulent strains (data not shown). Interestingly, Type I IFN (IFN-α) production was significantly induced in PPD-stimulated lung cells from Mtb H37Rv- and K-infected mice compared to Mtb Ra-infected mice, but it did not differ between Mtb H37Rv- and K-infected mice (Fig 5B).

### Functional properties of innate cells in the lungs of *M*. *tuberculosis*–infected mice upon interaction with T cells

**Fig 6:**
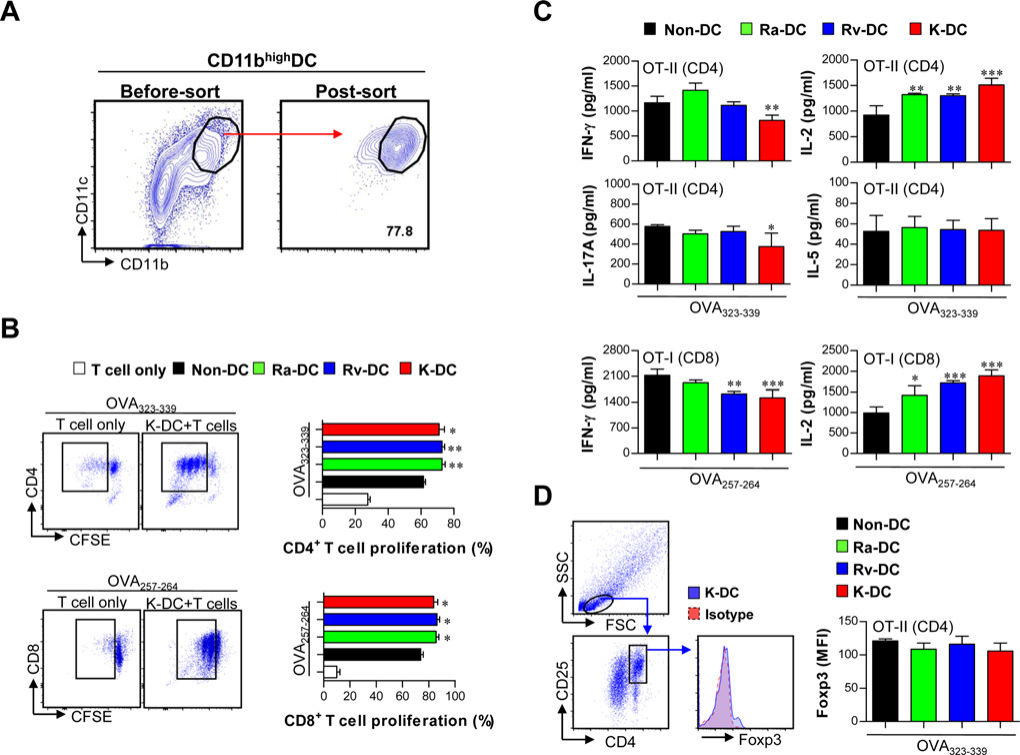
Analysis of T cell proliferation and polarization by CD11b^high^ DCs sorted from the lungs of Mtb-infected mice. (A) Specific populations of CD11b^high^ DCs were sorted from the lungs of mice infected with Mtb strains at 7 days post-infection. (B, C, D) CD11b^high^ DCs sorted from Mtb-infected mice were treated with OVA peptides (1 μg/ml), such as OVA_323-339_ or OVA_257-264_, for 1 h. The OVA-treated CD11b^high^ DCs were co-cultured with OVA-specific CD4^+^ and CD8^+^ T cells for 3 days at a sorted DC-to-T cell ratio of 1:10. (B) After 3 days of co-culture, the proliferation of OVA-specific CD4^+^ and CD8^+^ T cells was assessed by flow cytometry. (C) IFN-γ, IL-2, IL-5, and IL-17A were analyzed by ELISA. (D) CD25^+^Foxp3^+^ Treg cells were analyzed after co-culture with CD4^+^ T cells. The T cell data are shown as the mean ± SD (*n* = 5 samples). One representative plot out of two independent experiments is shown. *p < 0.05, **p < 0.01, and ***p < 0.001 compared with T cell/OVA-pulsed DCs sorted from non-infected mice. NI-DC: DC from non-infected mice; Ra-DC: DC from H37Ra-infected mice; Rv-DC: DC from H37Rv-infected mice; K-DC: DC from K-infected mice.

**Fig 7:**
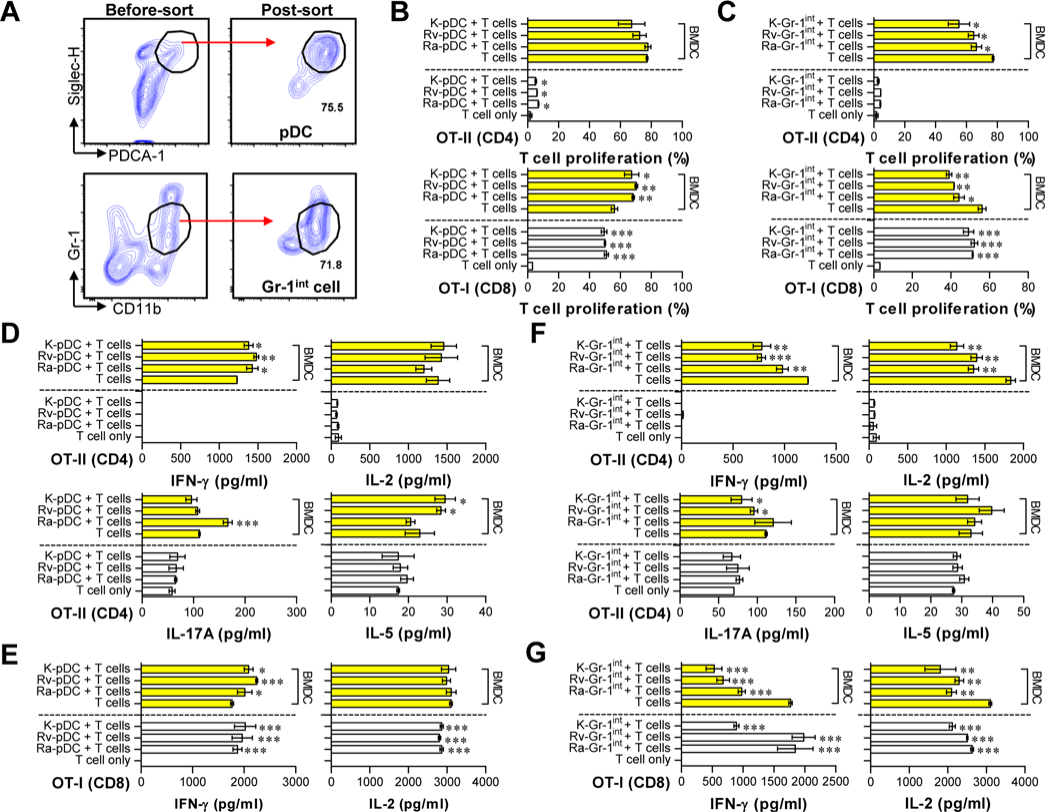
Analysis of T cell proliferation and cytokine generation induced by pDCs and Gr-1^int^ cells sorted from the lungs of Mtb-infected mice. Details regarding this experiment are provided in the materials and methods section. (A) Specific populations of pDCs and Gr-1^int^ cells were sorted from the lung cells of mice infected with Mtb strains at 28 days post-infection. T cell proliferation (B, C) and cytokine production (D-G) in response to pDCs and CD11c^-^/CD11b^+^/Gr-1^int^ cells with (yellow bars) and without (white bars) BMDCs were analyzed by flow cytometry after 3 days of co-culture with CFSE-labeled T cells. One representative plot out of two independent experiments is shown. *p < 0.05, **p < 0.01, and ***p < 0.001 compared with the T cell only or the BMDC + T cell group. Ra-pDC: pDC from H37Ra-infected mice; Rv-pDC: pDC from H37Rv-infected mice; K-pDC: pDC from K-infected mice; Ra-Gr-1^int^: Gr-1^int^ cells from H37Ra-infected mice; Rv-Gr-1^int^: Gr-1^int^ cells from H37Rv-infected mice; K-Gr-1^int^: Gr-1^int^ cells from K-infected mice.

Innate cells induced by Mtb infection can play an important role in the regulation of T cell immunity [25]. Thus, we investigated adaptive immunity induced by lung-infiltrating cells (CD11b^high^ DCs, pDCs and CD11c^-^CD11b^+^Gr-1^int^ cells) at 7 days and 28 days post-infection with Mtb strains (Fig 6 and Fig 7). We conducted a functional study with CD11b^high^ DCs, pDCs and CD11c^-^CD11b^+^Gr-1^int^ cells sorted from the lungs of Mtb strain-infected mice; we analyzed T cell proliferation, T cell polarizing cytokines (Th1: IFN-γ; Th2: IL-10 and IL-5; and Th17: IL-17A) and the induction of regulatory T cells (Tregs) using both OVA-specific CD8^+^ T cells from OT-I transgenic mice and CD4^+^ T cells from OT-II transgenic mice (Fig 6 and Fig 7). We verified that CD11b^high^ DCs sorted from Mtb-infected mice at 7 days post-infection induced significantly greater proliferation of both CD4^+^ and CD8^+^ T cells compared to CD11b^high^ DCs sorted from non-infected mice (Fig 6B). Regarding the induction of CD4^+^ and CD8^+^ T cell cytokines by sorted CD11b^high^ DCs, IL-2 secretion was increased by CD11b^high^ DCs sorted from K-infected mice compared to those sorted from non-infected mice, but IFN-γ and IL-17A secretion was decreased (Fig 6C). Additionally, IL-5 and Foxp3 levels in CD4^+^ T cells were not different (Fig 6C and 6D).

Next, pDCs sorted from Mtb-infected mice at 28 days post-infection directly induced the proliferation of CD4^+^ and CD8^+^ T cells (white bars in Fig 7B) and the production of IFN-γ and IL-2 in CD8^+^ T cells (white bars in Fig 7E), but there were no significant changes in IFN-γ, IL-2, IL-5 and IL-17A secretion from CD4^+^ T cells (white bars in Fig 7D). Although pDCs sorted from Mtb H37Ra-infected mice induced Th1 (IFN-γ) and Th17 (IL-17A) cytokines in T cells in the presence of BMDCs, pDCs sorted from Mtb H37Rv- and K-infected mice induced significantly greater levels of Th1 (IFN-γ) and Th2 (IL-5) cytokines compared to the BMDC + CD4^+^ T cell groups (yellow bars in Fig 7D). These results suggest that pDCs in mice infected with virulent Mtb strains may affect the maintenance of the Th2 response.

A recent study showed that CD11c^-^CD11b^+^Gr-1^int^ cells play a critical role in suppressing the T cell response [26]. Thus, we confirmed that CD11c^-^CD11b^+^Gr-1^int^ cells induced by Mtb infection can suppress the T cell response (Fig 7C, 7F and 7G). As a result, CD11c^-^CD11b^+^Gr-1^int^ cells sorted from Mtb-infected mice blocked the proliferation of and cytokine production by CD4^+^ and CD8^+^ T cells in the presence of BMDCs (yellow bars in Fig 7C, 7F and 7G), but there was no difference in the response of T cells to CD11c^-^CD11b^+^Gr-1^int^ cells in the absence of BMDCs (white bars in Fig 7C, 7F and 7G). Surprisingly, CD11c^-^CD11b^+^Gr-1^int^ cells sorted from Mtb H37Rv- and K-infected mice inhibited IL-17A production by T cells in the presence of BMDCs compared to the BMDC + CD4^+^ T cell groups (yellow bars in Fig 7F). However, the generation of Tregs by pDCs or CD11c^-^CD11b^+^Gr-1^int^ cells was not induced under any experimental condition (data not shown). These findings suggest that the high frequency of CD11c^-^CD11b^+^Gr-1^int^ cells induced by virulent Mtb infection may contribute to the down-regulation of both Th1 and Th17 responses.

## Discussion

Although there have been numerous studies on the functions of and alterations in immune cells during Mtb infection, only a few studies have used clinical Mtb isolates with highly virulent characteristics. In this study, we established an aerosol infection model in mice with different strains of virulent Mtb to investigate their differential pathogenesis in terms of bacterial growth and lung pathology. The Mtb K strain was the most virulent and was characterized by rapid growth at the early time points post-infection (by 28 days) and severe lung inflammation at the late time points (56–112 days). In contrast, Mtb H37Ra was the least virulent strain and did not reach optimal growth; its bacterial burden decreased after 28 days post-infection (Fig 1 and Fig 2). Next, we compared the alterations in innate cell components and helper T cell kinetics during lung infections with respect to the virulence of the Mtb strain in the mouse model. Importantly, in mice infected with Mtb K, there were virulence-dependent increases in Gr1^int^CD11b^+^ cells, pDCs and the Treg frequency as well as a decrease in the Th1 immune response over 28 days post-infection without the induction of a Th17 response. Interestingly, innate cell kinetics in the lungs of mice infected with Mtb H37Ra did not significantly differ from those of mice infected with Mtb H37Rv, except for pDC kinetics. Moreover, Mtb H37Ra induced high levels of both Th1-type and Th17-type T cells at later time points (112 days post-infection), while the Treg and Th2 responses decreased after 28 days post-infection. Mtb H37Rv exhibited intermediate virulence between Mtb K and Mtb H37Ra. A gradual increase in pDC and mixed Th1, Th2 and Th17 responses were features of the Mtb H37Rv-induced host response at late time points post-infection.

Virulence in TB pathogenesis in animal models is generally defined by the bacterial burden, bacterial survival via evading the host immune responses, and the capacity to cause severe lung damage [27].

The immunopathological environment in lung lesions can be manipulated by virulent Mtb strains by regulating the accumulated immune cells and the cytokine balance according to the infection stage [4, 28]. Clearly, progressive pulmonary TB is not a result of increasing numbers of viable bacilli in mice but rather a result of an excessive host response to virulent Mtb infection (Fig 1 and Fig 2). However, the types and functions of the accumulated innate cells are associated with the rapid initiation and maintenance of adaptive immunity. Thus, these cells are thought to be critical for successful immune protection before Mtb triggers pathogen-favored conditions [29, 30]. Furthermore, Mtb produces virulence factors, such as ESX-1 secretion-associated proteins, that regulate granuloma formation, dissemination, bacterial growth and the host immune response [31–33]. ESAT-6 and CFP-10 are secreted from Mtb by the ESX-1 (type VII) secretion system, which may contribute to the evasion of host immunity [34, 35]. Fredric *et al*. demonstrated that the ESX-1 secretion system is required for granuloma persistence and survival in immune cells [33]. Importantly, this system is present in virulent strains (Mtb H37Rv and Beijing strains) that are lacking the major part of the ESX-1 secretion system in attenuated other mycobacteria (H37Ra or BCG) [36, 37]. Our previous study showed that the ESAT-6 and CFP-10 antigens were more abundantly produced by Mtb K than by Mtb H37Rv [38]. Thus, the Mtb K strain may form pathogen-favored granulomas by altering host immune cell function and infiltration.

Consequently, although the innate cell populations that infiltrated in response to infection with three different Mtb strains were not significantly different at the early time points (<14 days post-infection) (Fig 3), CD11b^high^ DCs were strongly induced by infection with all the strains. Thus, we suspected that the function of DCs that infiltrated at the early time point may differ among Mtb strains. DCs recognize microbes and their products in mycobacterial-infected lung tissue and produce anti- or pro-inflammatory cytokines that regulate the development of T cell immunity [25]. As expected, CD11c^high^ DCs that infiltrated after Mtb K infection altered T cell responses towards decreasing Th1 and Th17 responses compared to naïve CD11c^high^ DCs at 7 days post-infection, but these effects were not observed after Mtb H37Ra or H37Rv infection (Fig 6). Given the reduced Th1 and Th17 responses in response to Mtb K-induced CD11c^high^ DCs, the types and responses of T cells at the late post-infection time point may generate pathogen-favored immune responses.

Although the relative contributions of each type and response of CD4^+^ helper T cell during different stages of infection remain poorly understood, these cells are involved in maintaining protective or pathogenic conditions in response to Mtb infection [5, 28, 39, 40]. Although the function of GATA-3-expressing Th2 cells in TB pathogenesis has been less emphasized recently, in this study, this type of T cell emerged more rapidly than Th1 cells, peaking at 14 *vs*. 28 days, respectively. Interestingly, GATA-3-expressing Th2 cells were maintained in the lungs of both Mtb K- and H37Rv-infected mice but were continually down-regulated in Mtb H37Ra-infected mice. Additionally, PPD-specific Th2 cytokines (IL-5 and IL-10) were consistently induced after Mtb K infection compared to Mtb H37Ra and H37Rv infection (Fig 5A). In addition, both virulent strains, Mtb K and H37Rv, induced T-bet-expressing Th1 cells at 14 days post-infection. However, this population was rapidly diminished in the lungs of Mtb K-infected mice beginning at 28 days post-infection. Ordway *et al*. also reported that the hypervirulent HN878 strain induced a potent Th1 response; however, Th1 immunity was rapidly down-regulated along with the emergence of a CD4^+^CD25^+^Foxp3^+^CD223^+^IL-10^+^ Treg population [41]. These results agree with those of our study. Although Mtb K induced T-bet-expressing Th1 cells, the PPD-specific Th1 cytokine (IFN-γ) was down-regulated, and high levels of Treg cells emerged after 56 days post-infection. Interestingly, Mtb H37Ra infection consistently induced T-bet-expressing Th1 cells and the PPD-specific Th1 cytokine, but this Th1 cell population increased in a time-dependent manner together with the down-regulation of CD4^+^CD25^+^Foxp3^+^ Tregs. It is well documented that Tregs decrease the ability of alveolar- and monocyte-derived macrophages to restrict the growth of Mtb in the presence of effector cells [42]. Although the mechanism of how virulent Beijing Mtb strains expand Tregs has not been studied to date, one possible explanation is that Beijing Mtb highly expresses Treg-specific antigens [43].

The reciprocal induction of Th1 and Th17 cellular responses has been shown to help establish protective immunity against TB [44]. Importantly, IL-17, TNF-α, and/or multi-functional T cells (IFN-γ^+^IL-2^+^TNF^+^ T cells) were observed at higher frequencies in sterile granulomas than in non-sterile granulomas [28]. In agreement with this previous report, H37Ra infection induced a greater reciprocal expansion of the Th1 and Th17 cell responses than Mtb H37Rv and K infection in our study.

Recently, the ability of type I IFNs to accelerate TB pathogenesis was revealed, which resulted in rapid mouse death with excessive lung pathology [45, 46]. Interestingly, a higher level of IFN-α was produced in lung cells from virulent Mtb strain-infected mice after PPD stimulation than in those from Mtb H37Ra-infected mice (Fig 5B). The pDCs, which are type I interferon (IFN)-producing cells [47], are the most interesting cell population correlated with the virulence of Mtb in this study. One intriguing clinical study examined the differences in the ratios of DC subsets in untreated and smear-positive pulmonary TB patients compared with healthy family contacts. The study indicated that a higher percentage of circulating pDCs than myeloid DCs (mDCs) is associated with active TB [48]. In addition, the ratio between mDCs and pDCs was restored after chemotherapy; non-responders to chemotherapy have more circulating pDCs than mDCs [48]. In this study, we showed that the frequency of pDCs increased in a virulence-dependent manner. Interestingly, the isolated pDCs from the lungs of mice infected with virulent Mtb strains significantly induced the simultaneous production of IFN-γ and IL-5 in the DC-CD4^+^ T cell co-culture system, whereas the pDCs from Mtb H37Ra-infected mice induced IFN-γ and IL-17A under the same culture conditions (Fig 7B, 7D and 7E). These results showed that pDCs generated by virulent Mtb strains may contribute to the simultaneous induction of Th1 and Th2 immune responses during their interactions with T cells. These characteristics were in agreement with the type of infiltrated helper T cells shown in Fig 4.

Another important population that increased in response to Mtb virulence is Gr1^int^CD11b^+^ cells. This population was recently recognized as being myeloid-derived suppressor cells (MDSCs) that are associated with TB pathogenesis [26, 49]. Recent studies have emphasized the role of Gr1^int^CD11b^+^ myeloid-derived suppressor cells, which have a similar cell surface marker to neutrophils, in Mtb pathogenesis [26, 49]. Tsiganov *et al*. reported that immature MDSCs, but not neutrophils, are markers of lethal TB infection in mice [49]. In this study, the neutrophil population in the lung did not differ among the tested Mtb stains, indicating that this population is not a reflection of the virulence of Mtb strains. Instead, Gr1^int^CD11b^+^ cells appear to accumulate in the lungs of Mtb-infected mice in a time-dependent and virulence-dependent manner, indicating that our results are in good agreement with those of the previous study. We also hypothesized that Gr1^int^CD11b^+^ cells generated by Mtb infection would have MDSC-like features, such as suppressing helper T cell immune responses. Consequently, the Gr1^int^CD11b^+^ cells that was isolated from the lungs of Mtb-infected mice at 28 days post-infection significantly decreased CD4^+^ and CD8^+^ T cell proliferation under DC-T cell co-culture conditions (Fig 7C). In addition, IFN-γ, IL-2 and IL-17A levels were significantly decreased in the presence of Gr1^int^CD11b^+^ cells generated by virulent Mtb infection (Fig 7F). Interestingly, these phenomenon were induced by Gr1^int^CD11b^+^ cells regardless of Mtb virulence. Thus, Gr1^int^CD11b^+^ cells may be a major cell type that suppresses helper T cell responses, and the increased frequency of Gr1^int^CD11b^+^ cells in the presence of virulent Mtb K may more strongly suppress protective T cell responses. Nevertheless, a better understanding of the presence and function of Gr1^int^CD11b^+^ cells during lung infection by Mtb, the immunologic consequences of direct interactions between Gr1^int^CD11b^+^ cells and Mtb and how Gr1^int^CD11b^+^ cells are generated all require further investigation.

In conclusion, our study characterized the kinetics and immune responses of the accumulated immune cells during Mtb infection according to strain virulence. Virulent Mtb strains may generate more pathogen-favored immune cells, such as pDCs, Gr1^int^CD11b^+^ cells and Tregs, to diminish the protective Th1 responses, thereby contributing to the inability of hosts to eradicate infection or mediating immunopathology in chronic infectious stages. In addition, the highly virulent Mtb K strain may trigger the combinatorial accumulation of pDCs to maintain an up-regulated Th2 response and of Gr1^int^CD11b^+^ cells to down-regulate the Th1 response, resulting in an immunopathological environment in lung lesions. Further investigations on which components of virulent Mtb manipulate the presenting cell phenotypes or the immunological environment may help us to understand how virulent Mtb evolved immunologically as the best-adapted human pathogen. It will also help us understand how TB manipulates the host immune system toward pathogen-favored conditions according to the infection stage.

## Supporting Information

S1 ARRIVE Checklist NC3Rs ARRIVE Guidelines Checklist. (PDF)

S1 Fig Representative histopathology of lungs infected with Mtb strains with different virulence at 28 and 56 days post-infection. Representative histopathology of lungs infected with Mtb strains with different virulence at 28 and 56 days post-infection. (A) Naïve, (B) Mtb H37Ra, (C) Mtb H37Rv, and (D) Mtb K.
(TIF)

S2 Fig Histopathology of lungs infected with the Mtb H37Rv or K strain at 112 days post-infection. (TIF)
