# Supplementary figures and images for: Virulence-Dependent Alterations in the Kinetics of Immune Cells during Pulmonary Infection by Mycobacterium tuberculosis
Source: PLoS One. 2015 Dec 16;10(12):e0145234. doi: 10.1371/journal.pone.0145234 (PMC4682951; doi:10.1371/journal.pone.0145234)

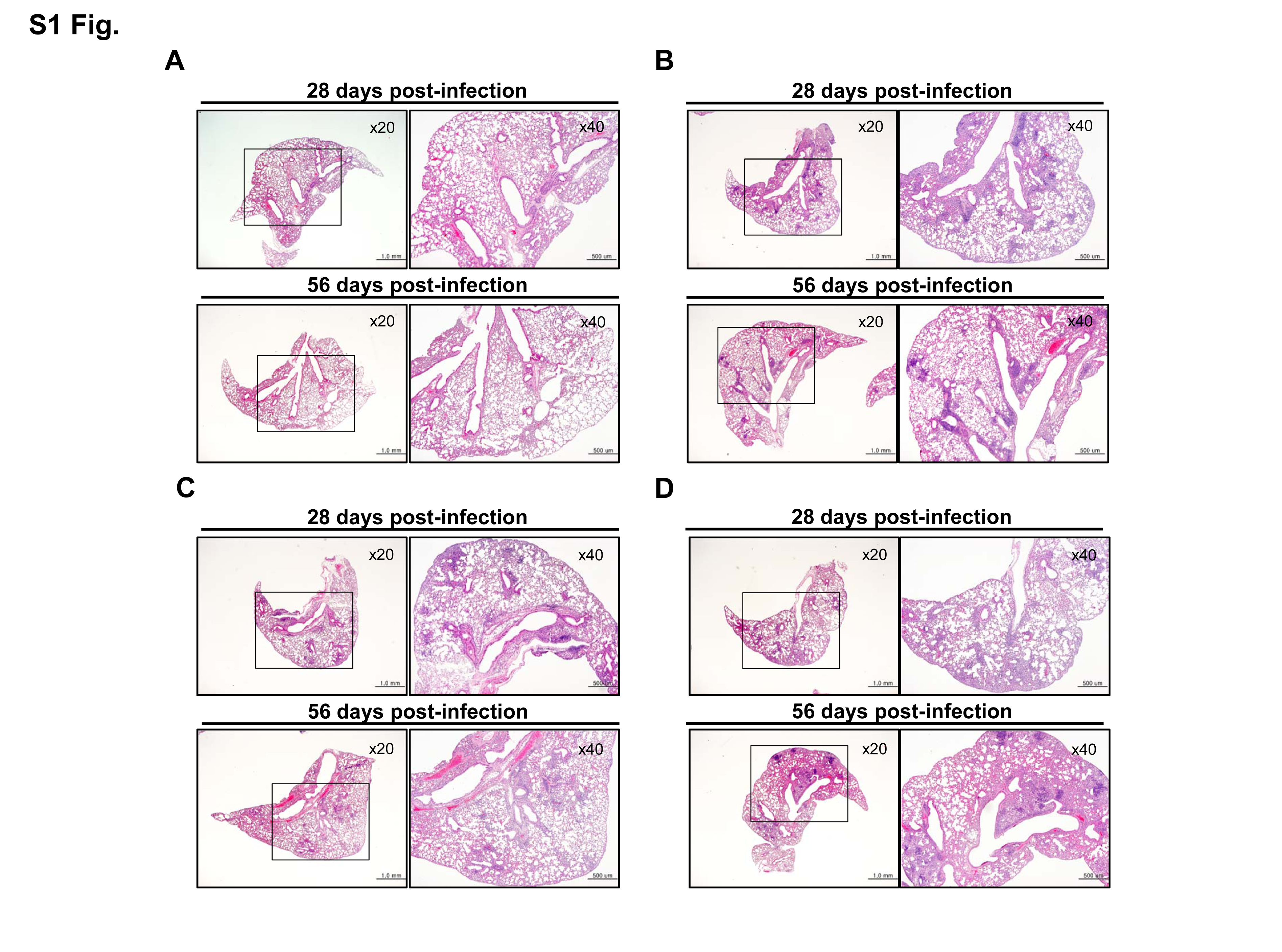

Supplement: S1 Fig — Representative histopathology of lungs infected with Mtb strains with different virulence at 28 and 56 days post-infection. (A) Naïve, (B) Mtb H37Ra, (C) Mtb H37Rv, and (D) Mtb K. (TIF) [file pone.0145234.s002.tif]

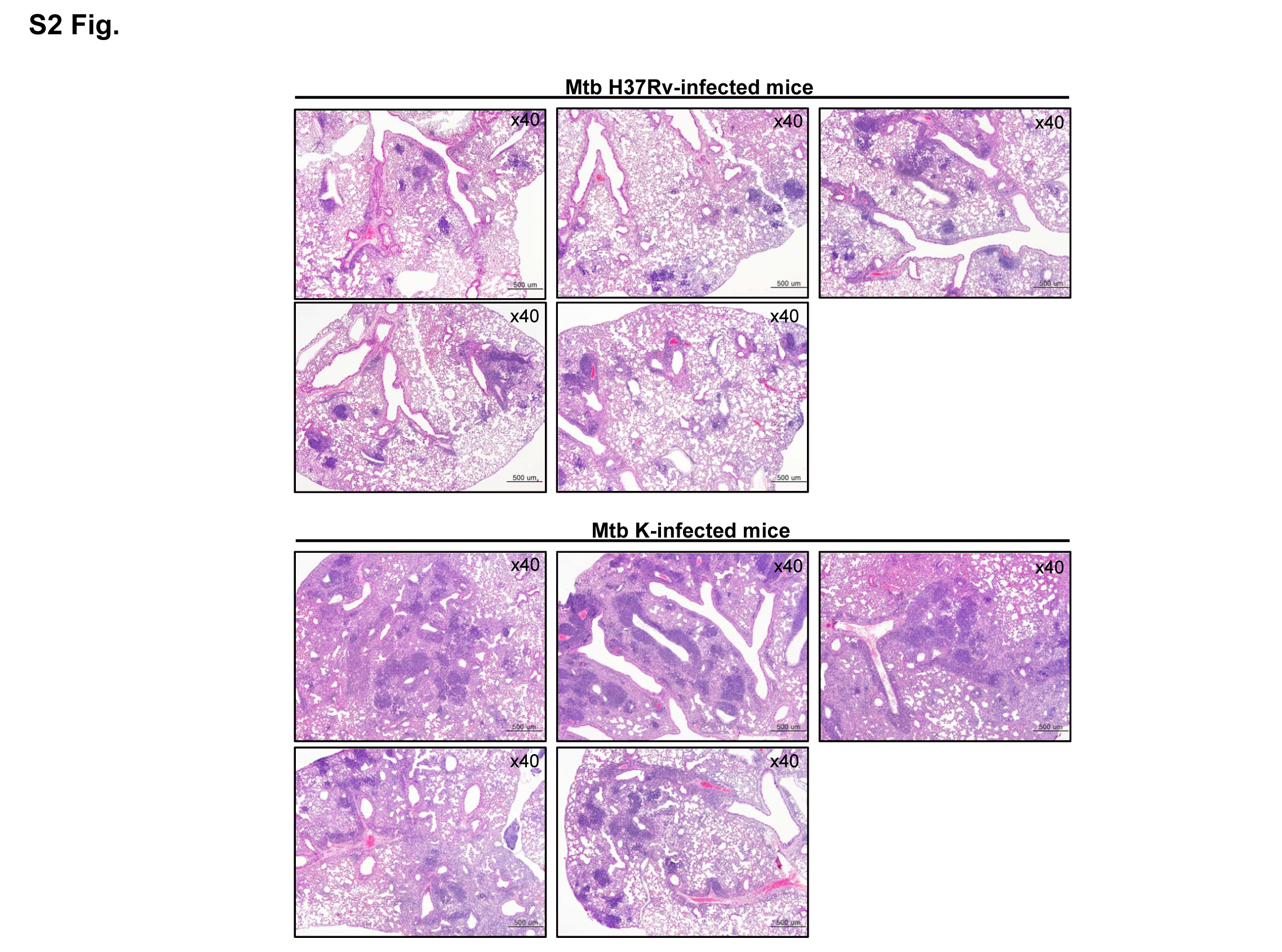

Supplement: S2 Fig — (TIF) [file pone.0145234.s003.tif]
